# Fat Grafting and Prepectoral Prosthetic Reconstruction with Polyurethane-Covered Implants: Protective Role against Adjuvant Radiotherapy

**DOI:** 10.3390/jcm13174982

**Published:** 2024-08-23

**Authors:** Liliana Barone Adesi, Federico Taraschi, Giulia Macrì, Lorenzo Scardina, Alba Di Leone, Gianluca Franceschini, Marzia Salgarello

**Affiliations:** 1 UO Chirurgia Plastica, Dipartimento per la Salute della Donna, del Bambino e di Sanità Pubblica, Fondazione Policlinico Universitario “Agostino Gemelli” IRCCS-Università Cattolica del “Sacro Cuore”-Largo A. Gemelli 8, 00168 Rome, Italy; liliana.baroneadesi@policlinicogemelli.it (L.B.A.); giulia.macri@live.it (G.M.); marzia.salgarello@policlinicogemelli.it (M.S.); 2 Breast Unit, Department of Women, Children and Public Health Sciences, Fondazione Policlinico Universitario “Agostino Gemelli” IRCCS-Università Cattolica del “Sacro Cuore”-Largo A. Gemelli 8, 00168 Rome, Italy; lorenzo.scardina@policlinicogemelli.it (L.S.); alba.dileone@policlinicogemelli.it (A.D.L.); gianluca.franceschini@policlinicogemelli.it (G.F.)

**Keywords:** prepectoral breast reconstruction, fat grafting, adjuvant radiotherapy

## Abstract

**Background/Objectives:** Breast cancer treatment increasingly incorporates immediate prepectoral prosthetic reconstruction after conservative mastectomy, including nipple-sparing (NSMs) and skin-sparing mastectomies (SSMs). Although recent data from the literature show that postmastectomy radiotherapy (PMRT) after prepectoral reconstruction presents good clinical results, with reduction in capsular contracture and implant migration, compared to the traditional submuscular technique, these patients have higher rates of long-term complications when compared with nonradiated patients. This study evaluates the protective effects of autologous fat grafting to reduce long-term radiotherapy-induced complications in breast cancer patients submitted for prepectoral reconstruction with polyurethane-covered (PU) implants. **Methods:** A pilot study with two parallel cohorts of patients undergoing an NSM or SSM followed by PMRT was conducted. Patients were randomly assigned to either of the two groups to ensure homogeneity. One cohort underwent autologous fat grafting sessions, individually tailored based on periodic evaluations by the principal investigator (PI), M. Salgarello, at least six months after PMRT. The control group received standard clinical follow-ups without fat grafting. Inclusion criteria ensured participants were disease-free, non-smokers, and had a LENT-SOMA score within 2. **Results:** Preliminary findings indicate significant differences between the groups, with improved outcomes observed in patients undergoing tailored lipofilling. Specifically, these patients experienced a notable reduction in capsular contracture severity and reported higher satisfaction with the aesthetic results compared to the control group. **Conclusions:** Autologous fat grafting, customized per patient by the PI based on ongoing evaluations, appears to mitigate some adverse effects of radiotherapy in prepectoral breast reconstruction, suggesting a viable option for enhancing surgical outcomes in irradiated patients. Further research is needed to substantiate these findings and evaluate long-term benefits.

## 1. Introduction

In recent years, immediate prepectoral breast reconstruction with silicone polyurethane-coated implants has been introduced [1,2,3,4]. These implants have long been established in both aesthetic and reconstructive settings. Since their introduction in 1968, rates of capsular contracture of 1–2% and great positional stability of the implant have been reported due to the rapid integration of the polyurethane foam, allowing the implant to be directly placed in the mastectomy pocket [5,6]. Since the implant requires no preparation or manipulation, operative times are reduced. The polyurethane coating is absorbed by the body and contributes to the formation of an ideal capsule that covers the implant, resulting in a soft and naturally appearing breast [1,2,3,4,5,6,7,8].

Adjuvant radiotherapy decreases the incidence of locoregional recurrences and increases survival in breast cancer patients. Post-mastectomy radiotherapy (PMRT) is indicated for patients with T3/T4 neoplasms, incomplete resection, and/or four or more positive axillary lymph nodes [9]. Recently, retrospective studies on locoregional recurrences after mastectomy, prospective clinical trials, and the latest meta-analysis by the Early Breast Cancer Trialists Collaborative Group have documented benefits of adjuvant radiotherapy even in patients with 1–3 positive lymph nodes. The Selective Use of Postoperative Radiotherapy after Mastectomy (SUPREMO) trial concluded with the publication of the latest results related to quality of life in 2018 [10]. Thus, a growing number of patients undergoing mastectomy may need to undergo PMRT in the near future.

Despite the oncological benefits, radiotherapy has a negative impact on surgical outcomes [7]. This is particularly true for submuscular-implant-based reconstructions, which increase the risk of complications and an aesthetically unsatisfactory result by more than four times [2]. Short-term complications include an increased risk of hematoma, seroma, wound dehiscence with prosthetic exposure, and infection. Long-term complications include resistance to expansion, chronic pain, capsular contracture, skin thinning that may result in the visibility of the prosthetic profile, deformation, and implant rupture [7].

Prepectoral breast reconstruction involves a lower rate of complications following PMRT compared to submuscular reconstruction; for example, the incidence of contracture is reduced (16% vs. 56%) and is of lesser degree, and there is less deformity of the reconstructed breast presumably due to the absence of the pectoralis major muscle covering the implant, whose radio-induced shortening causes the submammary sulcus to rise in the submuscular implant [11,12].

We already demonstrated in a previous paper that prosthetic reconstruction can be safely performed in selected radiated patients after multiple fat grafts and described the effects of fat grafting on capsular contracture [13]. In 2012, we highlighted how patients with a pre-treatment LENT-SOMA score of 1, after three sessions of breast adipose tissue grafting, had a resulting post-treatment LENT-SOMA score of 0, and they thus became candidates for prosthetic reconstruction [9,10,11,12,14,15,16,17,18].

In this context, no data are yet available in the literature on the efficacy of lipofilling in patients undergoing PMRT and prepectoral reconstruction with polyurethane-coated implants. The aim of this study is to evaluate the efficacy of this procedure in this specific category of patients.

## 2. Materials and Methods

This case–control study was designed to evaluate the protective role of autologous fat grafting in reducing long-term radiotherapy-induced complications in breast cancer patients. This study included two cohorts of women who underwent an NSM or SSM and prepectoral prosthetic reconstruction with polyurethane-coated implants followed by adjuvant radiotherapy. Patients were randomly assigned to either the fat grafting cohort or the control cohort, ensuring homogeneity between the groups. One cohort received autologous fat grafting starting six months post-radiotherapy, while the control cohort did not receive fat grafting but was monitored according to the same follow-up schedule.

Inclusion criteria were disease-free status, a minimum of six months post-radiation therapy, LENT-SOMA grade of 0–2, and non-smoking status or cessation of smoking at least three months prior to the fat grafting surgery. Exclusion criteria included current smoking or resumption post-surgery, positive oncological follow-up, and LENT-SOMA grade > 2. Patients presenting with more severe clinical symptoms, i.e., LENT-SOMA grade > 2, were preferentially referred for autologous reconstruction.

Patients were recruited from the Plastic Surgery Unit of the Policlinico Gemelli in Rome between 2019 and 2023. They were informed about the study objectives, procedures, potential risks, and benefits, and they provided written informed consent.

A total of 30 patients met the inclusion criteria for the fat grafting cohort. However, 8 patients were lost to follow-up, resulting in 22 patients in the fat grafting group. Similarly, 30 patients were initially included in the control cohort, with 3 patients lost to follow-up, resulting in 27 patients in the control group.

Fat grafting involved harvesting adipose tissue from patient-specific areas such as hips and inner thighs, using a blunt-tip cannula connected to a mild vacuum. The lipoaspirate was then processed by centrifugation at 3000 rpm for 5 s. The purified fat was injected into all quadrants of the irradiated breast using a multi-planar, multi-layered technique to ensure even distribution and integration.

Clinical outcomes were assessed at six months post-fat grafting and subsequently re-evaluated annually to determine the need for additional lipofilling based on an increase in the degree of capsular contracture and the presence of other symptoms such as discomfort/pain, local hardening, or progressive deterioration of the aesthetic result. If additional lipofilling was performed, patients were re-assessed six months post-operatively. Data on complications related to chronic radiation effects, such as capsular contracture, were documented using clinical assessments based on palpation and the established LENT-SOMA scale.

Data collection was executed solely by the principal investigator M. Salgarello to ensure consistency and homogeneity of data, as objective evaluation scales were not utilized. Collected data included demographic information, medical history, details of the surgical procedure, and clinical outcomes.

Patients underwent regular imaging studies, including ultrasound and MRI, to monitor the integrity of the implant and to verify the fate of the injected fat over time. Follow-up visits included physical examinations and patient-reported outcome measures (PROMs) to assess satisfaction and quality of life. All patients underwent oncological follow-up, and no recurrences of the disease were observed during the study period.

Statistical analysis compared incidence rates of capsular contracture and patient-reported outcomes between the two cohorts. Categorical data were analyzed using chi-square and Fisher’s exact tests, while continuous data were assessed using independent and paired *t*-tests. Significance was set at *p* < 0.05. All statistical analyses were performed using R software (Version 2024.04.2+764).

The study protocol was approved by the institutional review board (IRB) of the Policlinico Gemelli. This study was conducted in accordance with the principles of the Declaration of Helsinki and Good Clinical Practice guidelines. Informed consent was obtained from all subjects involved in this study.

## 3. Results

A total of 49 patients were included in this study, with 22 in the case group and 27 in the control group. Patients were selected based on specified inclusion and exclusion criteria and were monitored from 2019 to 2023 at the Plastic Surgery Unit of Policlinico Gemelli in Rome.

**Figure 1:**
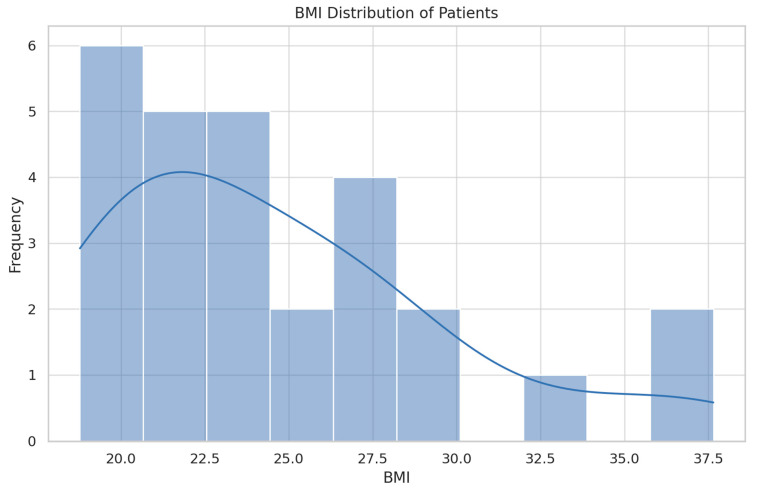
BMI distribution of patients.

**Figure 2:**
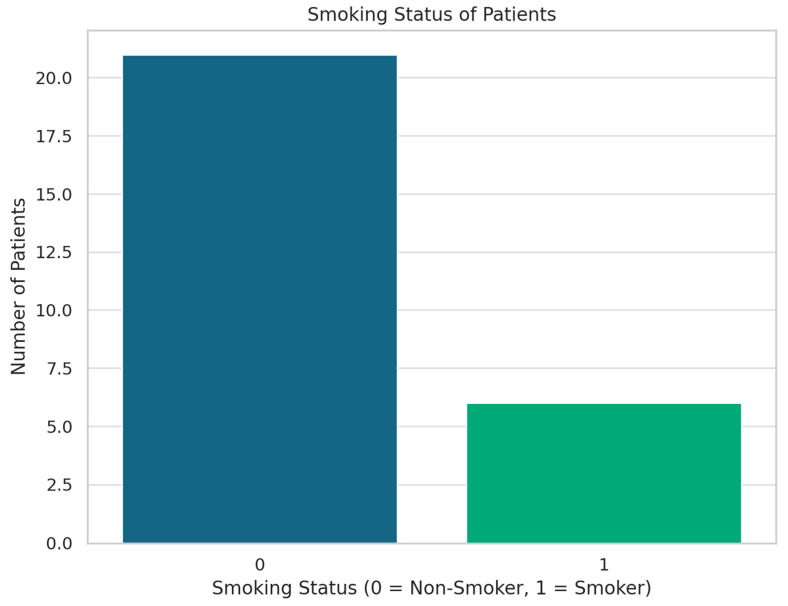
Smoking status of patients.

**Figure 3:**
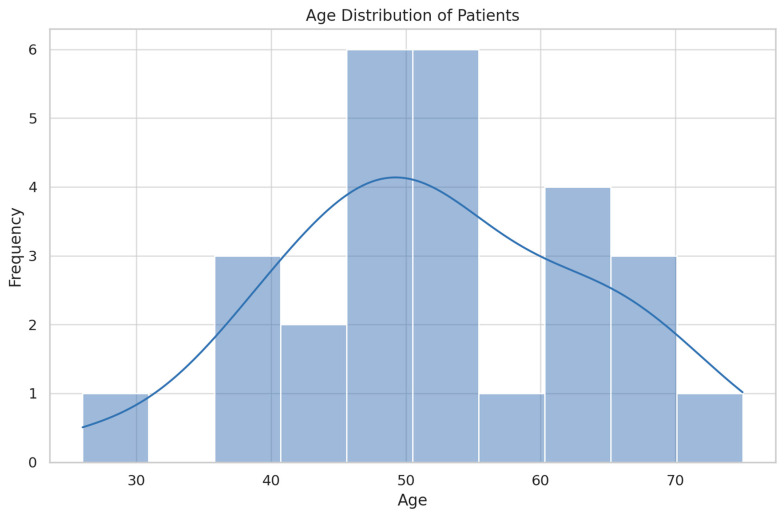
Age distribution.

The demographics of the study population are summarized in Table 1 and Figure 1, Figure 2 and Figure 3.

Both the cases and control groups were similar in terms of age, BMI, and smoking status, ensuring comparability between the two groups. This similarity is crucial for the validity of this case–control study, reducing potential confounding factors and supporting the reliability of the results.

**Figure 4:**
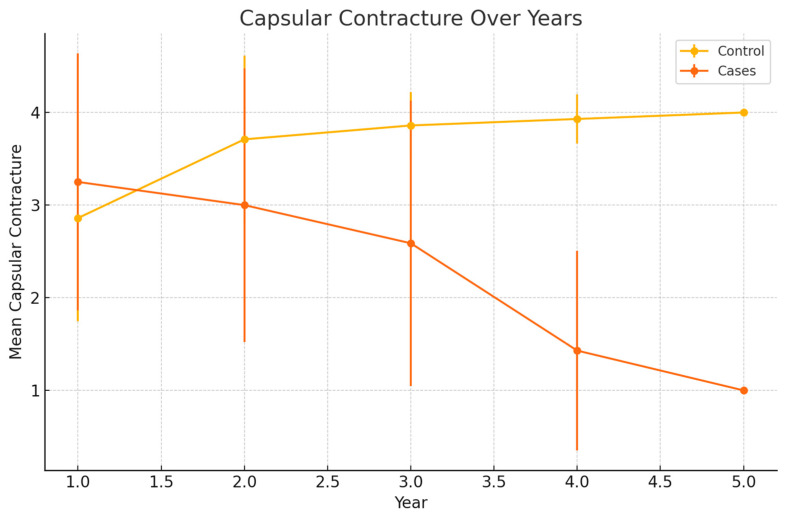
Capsular contracture over years.

Capsular contracture was assessed in both groups. In the case group, capsular contracture improved over time, decreasing from an average of 3.25 in the first year to 1.0 in the fifth year. In the control group, capsular contracture increased from an average of 2.86 in the first year to 4.0 in the fifth year (Table 2) (Figure 4).

**Figure 5:**
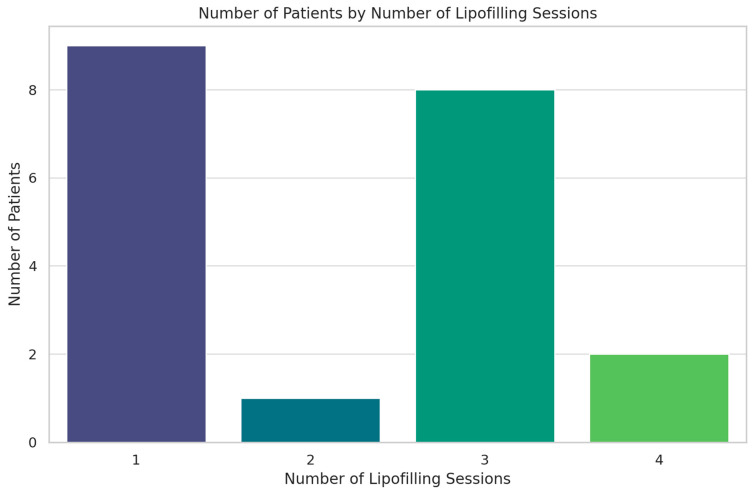
Number of patients by number of lipofilling sessions.

An analysis was conducted to investigate the correlation between the volume of lipofilling and the mean capsular contracture in patients. The results indicated that there is no significant correlation between the volume of lipofilling and capsular contracture (correlation coefficient = −0.012, *p*-value = 0.959). This suggests that the volume of lipofilling does not have a direct impact on the severity of capsular contracture. Additionally, the distribution of lipofilling volumes and the number of sessions per patient were analyzed. The volume of lipofilling carried out for each patient varied, with some patients receiving multiple sessions. The results are visualized in the accompanying figure (Figure 5).

**Figure 6:**
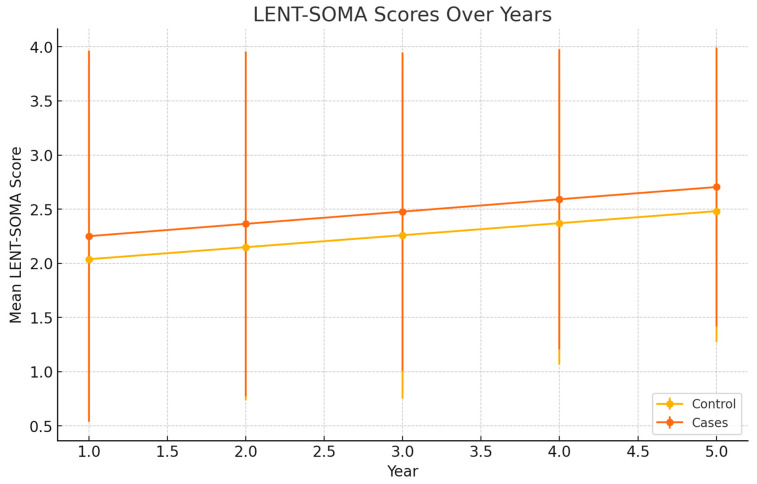
LENT-SOMA scores over years.

The LENT-SOMA scores were evaluated for both groups over the same period. The scores for the control group and case group showed no significant differences (Table 3) (Figure 6).

The statistical analysis compared the incidence rates of capsular contracture and LENT-SOMA scores between the two groups. Categorical data were analyzed using chi-square and Fisher’s exact tests, while continuous data were assessed using independent *t*-tests. Significance was set at *p* < 0.05.

To evaluate whether there were significant differences in capsular contracture between the control and case groups, independent sample *t*-tests were performed for each year. The *t*-test results showed significant differences in capsular contracture scores between the two groups for years 3, 4, and 5 (Table 4). Chi-square tests also showed significant differences for years 4 and 5 (Table 5).

To evaluate whether there were significant differences in LENT-SOMA scores between the control and case groups, independent sample *t*-tests were performed for each year. The *t*-test results showed no significant differences in LENT-SOMA scores between the two groups for each specific year (Table 6). Chi-square tests also showed no significant differences for each specific year (Table 7).

The case group showed significant improvement over time compared to the control group in terms of capsular contracture, suggesting that lipofilling may have a protective effect against capsular contracture in patients undergoing radiotherapy. However, there are no significant differences in LENT-SOMA scores between the two groups for each year, suggesting that lipofilling may not have a significant impact on this parameter. The statistical analysis supports these findings, with *t*-tests showing significant differences in capsular contracture scores for years 3, 4, and 5, and chi-square tests showing significant differences for years 4 and 5. For LENT-SOMA scores, both *t*-tests and chi-square tests showed no significant differences between the groups for each specific year.

## 4. Discussion

This present study investigated the effects of lipofilling on capsular contracture and LENT-SOMA scores in breast cancer patients undergoing prepectoral reconstruction with polyurethane-coated implants and subsequent PMRT. Our results demonstrated a significant improvement in capsular contracture over time in the case group undergoing autologous fat grafting sessions compared to the control group, suggesting a potential protective effect of lipofilling (Figure 4). However, no significant differences were observed in LENT-SOMA scores between the two groups (Figure 6).

Despite numerous studies on the subject, the pathophysiological mechanism of radio-induced capsular contracture remains unknown, although in submuscular reconstructions, it seems to be associated with the induction of muscle fibrosis that can cause upward displacement of the implant [14]. However, the effects of radiotherapy on healthy tissues are well known. In the literature, the different degrees of tissue damage observable in the first six months from exposure to radiotherapy are defined as acute, while after six months, radio-induced damage is classifiable as chronic. At the base of chronic tissue changes are microvascular damage and interstitial fibrosis. We believe that restoring healthy tissues after radio-induced damage can prevent complications associated with the placement of an implant. It has already been widely demonstrated that fat grafting is effective in revitalizing tissues with chronic radio-induced damage, promoting their biological and mechanical restoration. Rigotti et al. observed that three months after the grafting of autologous adipose tissue, the ultrastructural characteristics of the damaged tissue showed advanced regenerative phenomena [15].

Our study found that capsular contracture improved significantly in the case group over the five-year period, decreasing from an average of 3.25 in the first year to 1.0 in the fifth year. In contrast, the control group showed a worsening in capsular contracture, with an average increase from 2.86 in the first year to 4.0 in the fifth year. This suggests that lipofilling may have a protective effect against the development of capsular contracture in patients undergoing radiotherapy. These findings are consistent with those of previous studies that have suggested the benefits of fat grafting in reducing fibrotic reactions [19]. Previous studies have indicated that fat grafting can reduce fibrosis and improve tissue quality in irradiated areas [13,20,21,22,23]. Our results align with these findings, supporting the hypothesis that lipofilling has a beneficial effect on capsular contracture.

However, our study found no significant differences in LENT-SOMA scores between the case and control groups, which is apparently at odds with data highlighting the properties of lipofilling in ameliorating radiation-induced soft tissue damage. It is the authors’ opinion that the LENT-SOMA scale represents a rather crude index for determining clinical radiation damage, which therefore cannot capture minor clinical variations. Our findings suggest that it may be necessary to develop a more sensitive clinical index to assess radiation damage.

Our results suggest that lipofilling may be a valuable adjunctive therapy for patients undergoing PMRT, potentially reducing the severity of capsular contracture. Our data, which pertain to prepectoral breast reconstruction with polyurethane-coated implants, indicate the potential benefits of this approach.

There are several limitations to our study that should be considered. Firstly, the sample size was relatively small, which may limit the generalizability of our findings. Secondly, the follow-up period, although spanning five years, may still be insufficient to observe long-term outcomes of lipofilling. Additionally, this study relied on subjective assessment scales for evaluating capsular contracture, which may introduce biases and variability in the measurements.

## 5. Conclusions

Clinicians should consider incorporating lipofilling into the treatment regimen for these patients, although further research is needed to establish standardized protocols and confirm the long-term benefits.

Future studies should aim to include larger sample sizes and longer follow-up periods to confirm the protective effects of lipofilling against capsular contracture.

## Figures and Tables

**Table 1:** Summary of patient demographics.

Metric	Age	BMI	Smoking Status
Mean	52.15	24.60	
Standard Deviation	11.58	5.08	
Minimum	26	18.78	
25th Percentile	46	20.96	
Median (50th Percentile)	51	23.53	
75th Percentile	61	26.92	
Maximum	75	37.65	
Non-Smokers (0)			21
Smokers (1)			6

**Table 2:** Mean capsular contracture over time.

Year	Group	Mean Capsular Contracture	Standard Deviation
1	Control	2.86	1.112
1	Cases	3.25	1.389
2	Control	3.71	0.902
2	Cases	3.0	1.477
3	Control	3.86	0.363
3	Cases	2.588	1.543
4	Control	3.93	0.267
4	Cases	1.429	1.076
5	Control	4.0	0.0
5	Cases	1.0	0.0

**Table 3:** Mean LENT-SOMA scores over time.

Year	Group	Mean LENT-SOMA Score	Standard Deviation
1	Control	1.89	1.315
1	Cases	2.00	1.713
2	Control	2.148	1.412
2	Cases	2.364	1.591
3	Control	2.259	1.509
3	Cases	2.477	1.470
4	Control	2.370	1.306
4	Cases	2.591	1.388
5	Control	2.481	1.210
5	Cases	2.704	1.289

**Table 4:** *t*-test results for capsular contracture.

Year	Test Statistic	*p*-Value
1	−0.9463	0.3507
2	−1.5320	0.1356
3	−2.9370	0.0054
4	−5.9677	0.0000
5	−7.9372	0.0000

**Table 5:** Chi-square test results for capsular contracture.

Year	Chi-Square Value	*p*-Value
1	0.025	0.8759
2	0.401	0.5269
3	2.086	0.1489
4	7.609	0.0058
5	11.764	0.0006

**Table 6:** *t*-test results for LENT-SOMA scores.

Year	Test Statistic	*p*-Value
1	−0.4639	0.6457
2	−0.4768	0.6341
3	−0.4861	0.6273
4	−0.4963	0.6200
5	−0.5041	0.6137

**Table 7:** Chi-square test results for LENT-SOMA.

Year	Chi-Square Value	*p*-Value
1	0.0106	0.9181
2	0.0103	0.9190
3	0.0100	0.9202
4	0.0098	0.9210
5	0.0096	0.9220

## Data Availability

The data supporting the findings of this study are not available due to privacy and confidentiality concerns.
